# Increasing lean muscle mass: nutritional and periodization strategies

**DOI:** 10.1186/2052-1847-7-S1-O9

**Published:** 2015-08-11

**Authors:** James P Morton

**Affiliations:** 1 Research Institute for Sport and Exercise Sciences, Liverpool John Moores University, Liverpool, L3 3AF, UK

## 

Increases in skeletal muscle mass arises as a result of positive net protein balance, such that muscle protein synthesis (MPS) exceeds muscle protein breakdown (MPB). Increased mechanical loading and provision of high quality amino acids are potent and independent stimulators of MPS through activation of key cell signaling pathways involving the mTOR-p70S6K signaling axis. Importantly, the combination of resistance exercise and dietary protein intake elevate MPS over and above their independent effects, thus highlighting that a well formulated resistance training programme (incorporating multiple sets to failure) and increased dietary protein intake (spread evenly throughout the day) should form the basis of training and nutritional strategies. However, given that many elite athletes (such as rowers) may simultaneously be training to develop endurance as well as strength, it is also important to consider the periodization of endurance type training sessions alongside high volume resistance sessions. In this regard the signaling pathway known to regulate the endurance phenotype (i.e. the AMPK-PGC-1 axis) may potentially attenuate the activation of growth related pathways thereby mediating a training interference effect by which lean mass growth is negated. As such, careful consideration should also be given to the training structure and nutrient availability both within and between days so as to maximize training adaptation and recovery. In this presentation, the author presents data from both his own laboratory and others that briefly outlines the molecular regulation of muscle mass growth and endurance adaptations before providing nutritional and training periodization strategies such that both aspects of training adaptation may be developed simultaneously.

